# Two Novel NYX Gene Mutations in the Chinese Families with X-linked Congenital Stationary Night Blindness

**DOI:** 10.1038/srep12679

**Published:** 2015-08-03

**Authors:** Shuzhen Dai, Ming Ying, Kai Wang, Liming Wang, Ruifang Han, Peng Hao, Ningdong Li

**Affiliations:** 1 Henan Eye Institute, Henan Eye Hospital, Henan, People’s Republic of China; 2 Tianjin Key Lab of Ophthalmology and Visual Science, Tianjin Eye Institute, Tianjin, People’s Republic of China; 3 Tianjin Eye Hospital, Clinical College of Ophthalmology, Tianjin Medical University, Tianjin, People’s Republic of China

## Abstract

Mutations in *NYX* and *CACNA1F* gene are responsible for the X-linked congenital stationary night blindness (CSNB). In this study, we described the clinical characters of the two Chinese families with X-linked CSNB and detected two novel mutations of c. 371_377delGCTACCT and c.214A>C in the *NYX* gene by direct sequencing. These two mutations would expand the mutation spectrum of *NYX.* Our study would be helpful for further studying molecular pathogenesis of CSNB.

Congenital stationary night blindness (CSNB) is a group of clinically and genetically heterogeneous retinal disorders characterized by night blindness, decreased visual acuity, and a reduced or absent b-wave in the electroretinogram (ERG)^1^. Other clinical features of CSNB may include variable degrees of myopia, a nearly normal fundus appearance, nystagmus and strabismus. Two subgroups of CSNB can be classified by ERG into the “complete form” (or type 1 CSNB), and “incomplete form” (or type 2 CSNB)^2^. The complete form is characterized by absence of rod b-wave and oscillatory potentials due to complete loss of the rod pathway function, whereas the incomplete form shows a reduced rod b-wave, cone a-wave, and 30-Hz flicker ERG response caused by impaired rod and cone pathway function^3^.

CSNB may be inherited as an autosomal dominant, autosomal recessive and X-linked inheritance mode. Up to date, mutations in 14 genes, including *CACNA1F* (MIM300110), *NYX* (MIM 300278), *TRPM1* (MIM 603576), *LRIT3* (OMIM, 615004), *GRM6* (MIM 604096), *GPR179* (MIM 614515), *CABP4* (MIM 608965), *GNAT1* (OMIM, 139330)*, PDE6B*(OMIM, 163500) *RHO*(OMIM, 180380)*, GRK1* (OMIM, 180381)*, RDH5*(OMIM, 136880)*, SAG*(OMIM, 181031), and *SLC24A1*(OMIM, 603617) (Retnet:  http://www.sph.uth.tmc.edu/retnet/), have been reported to be responsible for CSNB. These genes are involved either in different components of the phototransduction cascade or in signaling transduction from photoreceptors to adjacent bipolar cells^4^.

*NYX* and *CACNA1F* genes are two disease-causing genes for X-linked congenital stationary night blindness. Mutations in *NYX* gene could be responsible for 45% of the X-linked CSNB, and may lead to the complete form of CSNB (CSNB1A), whereas mutations in *CACNA1F* gene could explain 55% of the X-linked CSNB and result in the incomplete form of CSNB (CSNB2A)^4^.

Here we reported two Chinese families with X-linked CSNB. Molecular genetic analysis of the two candidate genes (*NYX* and *CACNA1F*) indicated that two novel mutations in the *NYX* were detected in these two families.

## Materials and Methods

### Patient Ascertainment

**Figure 1:**
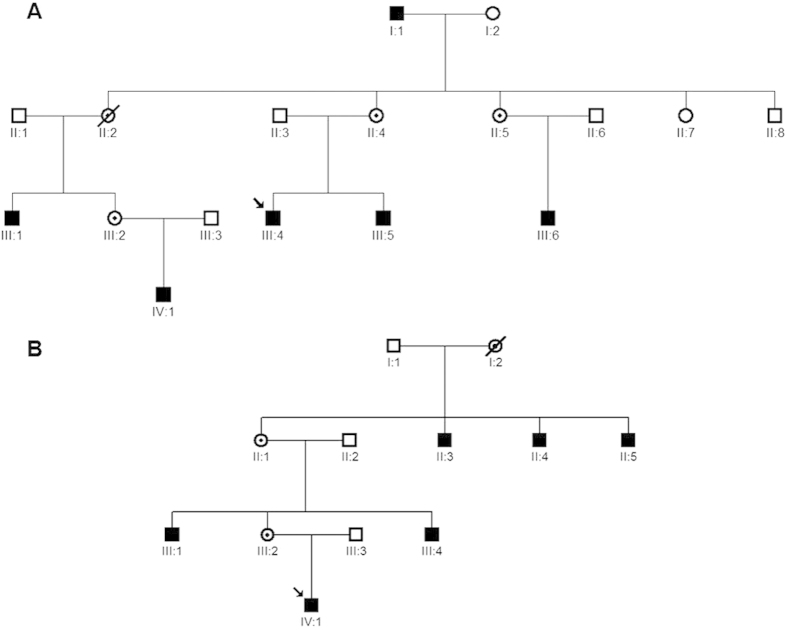
Two Chinese families with X-linked Congenital Stationary Night Blindness. The family CSNB-01 consisted of 6 affected males (individuals I:1, III:1, III:4, III:5, III:6, IV:1) and 3 female carrier (II:4, II:5, III:2), in which the individual III:4 was the proband (**A**) The family CSNB-02 included 6 affected males (individuals II:3, II:4, II:5, III:1, III:4, IV:1) and 2 female carriers (II:1, III:2). The individual IV:1 was the proband. (**B**).

Two families with X-linked CSNB (CSNB-01, and CSNB-02) were recruited for this study (Fig. 1). All participants underwent an ophthalmologic and orthoptic examination, including best corrected visual acuities, visual field, anterior segment of the eyes, vitreous and fundus, measurement of the deviation angle in the cardinal eye positions, examination of ocular movement and binocular vision, and Electroretinography (ERG). ERG were recorded according to the standards of the International Society for Clinical Electrophysiology of Vision (http://www.iscev.org). Cycloplegic refraction were performed for children under the age of 14. The spherical equivalent of the refractive error is given in diopters (D). After informed consent, 7 affected and 13 unaffected individuals from the two unrelated families were taken 3 ml blood samples from their blood vessels and DNA was extracted from blood lymphocytes according to the standard methods of protocol (Roche Biochemical, Inc). This study obtained IRB approval from the Tianjin Eye Hospital and conformed to the tenets of the Declaration of Helsinki.

### Mutation Analysis

Because *NYX* and *CACNA1F* are two candidate genes for X-linked congenital stationary night blindness, both of these two genes were directly sequenced in the CSNB-01 and CSNB-02 families. The coding regions and exon-intron boundaries of these two genes were amplified by PCR reaction using the primer pairs designed by the online software of the Primer 3. The exon 3 of *NYX* gene was split into five overlap fragments. The primer sequences were listed in the supplement Table. PCR were carried in 20 μL of standard PCR buffer containing 1.5 mM MgCl_2_, 0.2 mM of each dNTP, 0.5 μM of each primer, 1 U of Taq polymerase (Sangon, Shanghai, China), and 50 ng of DNA. The amplification program was an initial 2min denaturation at 98 °C, followed by 30 cycles of 30 s at 94 °C, 30 s at 55 °C, 1 min at 72 °C, and a final 7 min extension step at 72 °C. The PCR products were bi-directionally sequenced using the BigDye Terminator Cycle Sequencing V3.1 kit on an ABI PRISM 3130 Genetic Analyzer (Applied Biosystems) after purification with the QIAquick Gel Extraction Kit (Qiagen, Valencia, CA). Sequencing results were assembled and analyzed with the Seqman program of DNASTAR software (DNASTAR Inc, Madison, WI). The reference cDNA sequences of the *NYX* and *CACNA1F* were obtained from Genebank (NM_022567 for *NYX* mRNA, and NM_001256789 for *CACNA1F* mRNA) and +1 corresponds to the A of the ATG translation initiation codon. Mutation naming followed the nomenclature recommended by the Human Genomic Variation Society (HGVS).

#### In silico analysis

Two online programs of Polymorphism Phenotype (PolyPhen) and Sorting Intolerant From Tolerant (SIFT) were used to predict the potential impact of an amino acid substitution on the function of the protein. The molecular model were built using Swiss-model^5^,^6^,^7^,^8^.

## Results

### Clinical features

**Figure 2:**
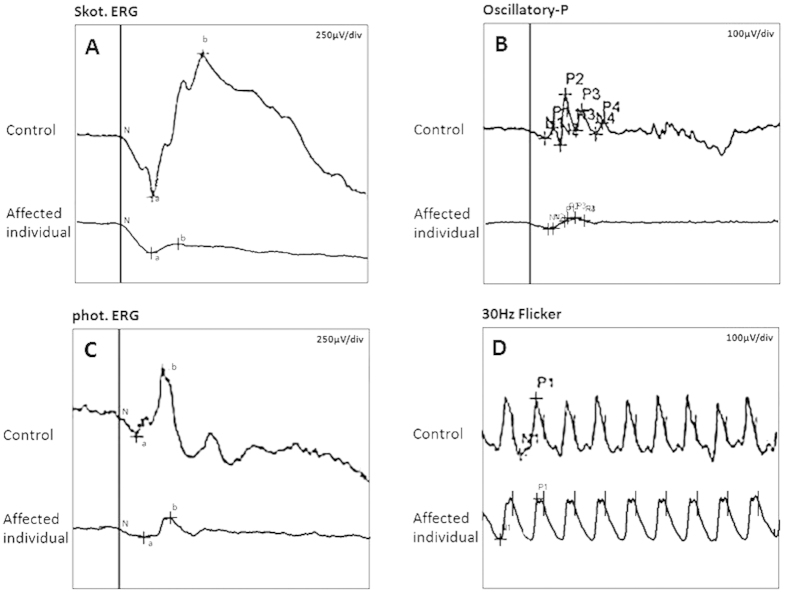
ERG recordings for the right eye of the proband and a normal control (individual III:3) in the family CSNB-01. Comparing with the normal control, the affected individual had abnormal ERG responses showing absence of b-wave under scotopic condition (**A**) loss of the wavelets of the oscillatory potentials (**B**) a normal photopic ERG (**C**) and 30 Hz flicker response (**D**).

In the family CSNB-01, all affected males complained about night blindness since their early childhood. Ophthalmologic examination showed that the patients had decreased visual acuity, variable degrees of myopia, latent nystagmus, and extropia combined with dissociated vertical deviation (DVD). Their detail clinical data were listed in the table 1. The proband (individual III:4, Fig. 1A) was a 24-year-old affected male and feel night blindness at the age of 4. Fundus examination revealed no obvious anomalies. Refraction examination showed a refractive error of −6.25D in his right eye and of −6.0D in his left eye. The best corrected visual acuity of his both eyes were 0.4. He had an extropia of about 15° at the primary eye position, and had fairly pronounced latent nystagmus combined with dissociated vertical deviation. The ERG showed absence of rod b-wave and oscillatory potentials under the scotopic condition, but a normal 30 HZ flicker response (Fig. 2). Thus, the clinical phenotype of the family CSNB-01 could be classified as the complete form of CSNB according to the ERG. The CSNB-02 family consisted of 6 affected males. The proband of the CSNB-02 family (individual IV:1, Fig. 1B) was a 7-year-old affected boy. He was diagnosed with CSNB by inquiring family history because his two uncles (individual III:1,III:4) were diagnosed with CSNB in other hospital. Refraction examination showed a refractive error of −9.0D sph in his right eye and of −10.0D sph in his left eye. The best corrected visual acuity of both eyes were 0.7. He had an intermittent extropia of 30 prism diopter (PD) at distance and 40 PD at near fixation. However, nystagmus and DVD were unremarkable. Fundus examination were high myopia changes but were otherwise normal. ERG examination was failed because he cannot cooperate very well. The CSNB form were also not able to established without ERG.

### Identification of Mutations

**Figure 3:**
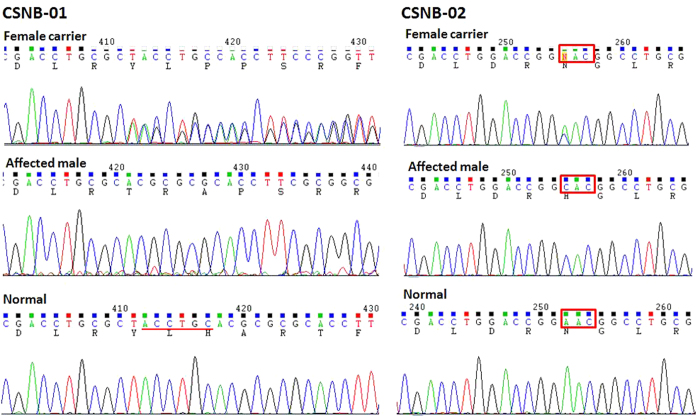
Sequence analysis of *NYX* gene for two families with X-linked CSNB. Sequencing chromatograms from a female carrier (top), an affected male (middle) and a normal individual (bottom) in each family. Mutations in *NYX* were identified in each of two families: c. 371_377delGCTACCT in the family CSNB-01 and c.214A>C in the family CSNB-02.

**Figure 4:**
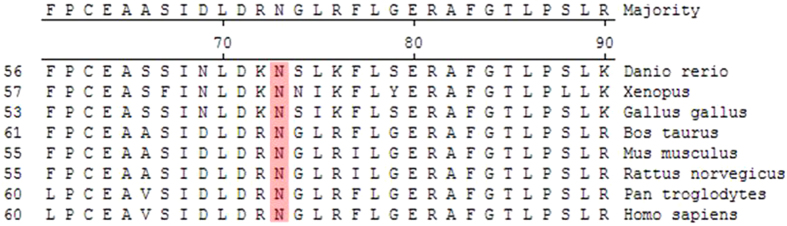
Multiple sequence alignment of the NYX protein. Multiple alignment of amino acids around p.N72H (denoted by the black framework) revealed evolutionary conservation of the Asparagine among Homo sapiens, Pongo abelii, Rattus norvegicus, Mus musculus, Box Taurus, Gallus gallus, Xenopus, and Danio rerio.

After sequence analysis to the *NYX* and *CACNA1F* genes, a 7-base pair deletion of c. 371_377delGCTACCT(p.Y125TfsX138) was detected in exon 3 of *NYX* gene in all affected males and female carriers in the family CSNB-01, while in the same exon, a missense mutation of c.214A>C(p.N72H) was identified in the family CSNB-02 (Fig. 3). Multiple sequence alignment of the NYX protein shows that N72 is conserved among Homo sapiens, Pongo abelii, Rattus norvegicus, Mus musculus, Bos taurus, Gallus gallus, Xenopus, and Danio rerio (Fig. 4 ). A PISC score of 1.0 and a SIFT score of 0.02 were yielded after using the POLYPHEN 2 (http://coot.embl.de/PolyPhen/) and SIFT (http://sift.bii.a-star.edu.sg/) program to predict the functional and structural changes of the amino acid substitution, which means that the substitution of Asparagine (Asn, N) at codon 72 by Histidine would be deleterious to the structure and function of the NYX protein. The above two mutations in *NYX* were absent in 100 normal controls after sequencing of the *NYX*.

## Discussion

*NYX* gene is located on chromosome Xp11.3 and consists of 3 exons spanning approximately 28 kb. It is mainly expressed in the kidney and retina. Within the retina, *NYX* is expressed in the inner segment of photoreceptors, outer and inner nuclear layers and the ganglion cell layer^9^. Nyctalopin is the encoded protein of the *NYX* gene, and consists of 481 amino acids. Structurally, nyctalopin belongs to a member of the leucine-rich repeat (LRR) superfamilies, containing 11 consecutive LRRs flanked by N- and C-terminal cysteine-rich LRRs, as well as an N-terminal ER signal peptide and a C-terminal glycosylphosphatidylinositol (GPI) membrane anchor^10^. LRRs are short-sequence motifs presenting in a number of proteins and are believed to be involved in the protein–protein interactions, cell adhesion and axon guidance^11^,^12^,^13^. Up to date, more than 50 mutations in *NYX* have been reported to be associated with congenital stationary night blindness. These mutations include missense and nonsense, splicing site mutations, as well as insertions and deletions. However, the exact molecular pathogenesis of CSNB caused by *NYX* mutations remains to be elucidated. It was suggested that nyctalopin might play an important role in keeping localization of TRPM1 in the dendritic tips of ON bipolar cells^14^,^15^. As an accessory subunit of the transient receptor potential (TRP) channel, nyctalopin might interact directly with TRPM1, or together with LRIT3 (another LRR family member), to keep the signaling channel proteins in the right place. The dislocation of TRPM1 would lead to abnormal function of retinal depolarizing bipolar cells (DBCs) and be predicated to result in abnormal signal transmission from photoreceptors. Mutations in these three interacted proteins (Nyctalopin, LRIT3 and TRPM1) have been documented to be associated with the complete form CSNB.

The mutation of c. 371_377delGCTACCT(p.Y125TfsX138) is located in exon 3 of the *NYX* gene. The 7 base pair of GCTACCT deletion would result in a frameshift change of *NYX* gene and produce a truncated proteins with initial 125 amino acids similar to the wild type NYX protein and additional 12 aberrant amino acids followed by a premature stop codon. Thus, the NMD surveillance mechanism could be activated by a PTC-containing mRNA. The abnormal mRNA with PTC would be degraded under the NMD surveillance mechanism or produce a defective truncated protein through escaping the NMD surveillance^16^,^17^.

**Figure 5:**
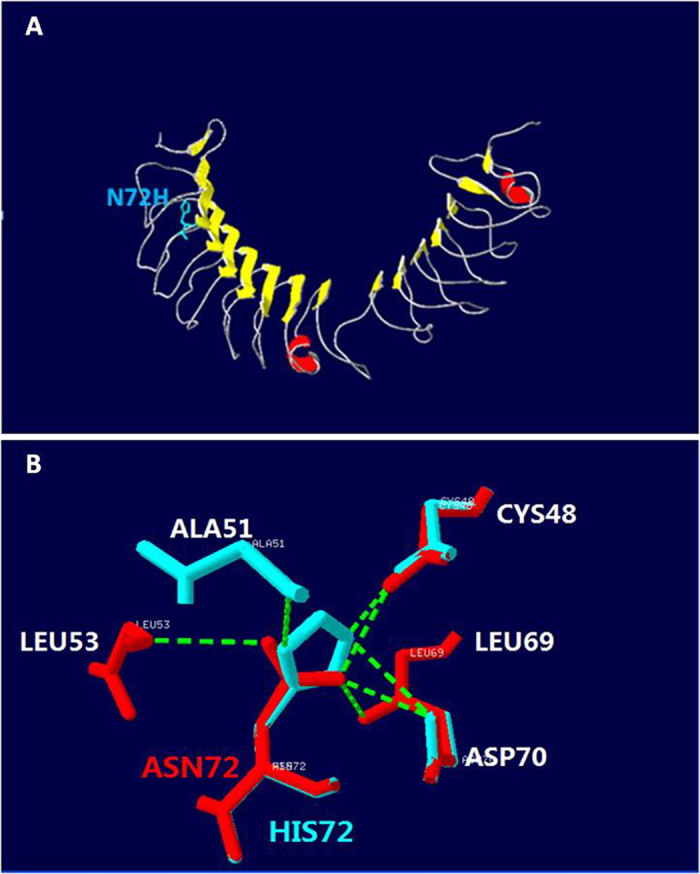
Structural model of nyctalopin built by the Swiss model. (**A**) 3-Dimensional structure of nyctalopin. Yellow arrows show beta-strands. Red parts show α-helix. The side chains of N72 are marked in blue. (**B**) Detail of the effect of the N72H mutation showing loss of hydrogen bonds. The normal and mutated nyctalopin structures are shown in red and blue, respectively. Hydrogen bonds are shown by green dashed solid lines.

Mutation of c.214A>C (p.N72H) in the family CSNB-02 is a novel mutation and not detected in 100 normal controls. N72 is located in the first repeat of leucine-rich repeat (LRR) in the nyctalopin, and is highly conserved among human and the lower animals. Substitution of Asparagine by Histidine would be probably damaging to the protein structure and/or the protein function, predicted by either POLYPHEN 2 or SIFT program. In addition, Asparagine 72 forms the inter-side chain hydrogen bonds with Cysteine 48, Leucine 53, Leucine 69 and Aspartate 70. Substitution of Asparagine by Histidine would break hydrogen bonds with Leu 53 and Leu 69, and forms a new hydrogen bond with Alanine 51 in the Swiss-model (Fig. 5), which could be expected to change the protein structure. However, changing Asparagine to Histidine were not able to disrupt the concave exterior surface of the secondary molecular structure in the constructed model as to be expected (Fig. 5). Previous study documented that different constructs containing *NYX* mutations could not produce any effect on the cellular localization in comparison to wild type constructs^18^, and the mutated variants in the LRR might cause CSNB through disrupting the protein-protein interactions^11^,^19^. The detailed molecular mechanism should be elucidated further by *in vitro* and *in vivo* experiments.

Although more than 50 mutations in *NYX* have been reported and could be responsible for 45% of the X-linked CSNB, only three *NYX* mutations, including p.Arg94Pro^20^, p.Leu184Arg^20^ and p.Thr258Pro^21^, have been reported in the Chinese patients with CSNB. Mutations of p.Arg94Pro and p.Leu184Arg are located in the second and the third LRR motif respectively, whereas mutation of p.Thr258Pro located in the ninth LRR motif. To our knowledge, mutation of N72H in the first LRR motif have not been reported before. With exception of the CSNB, *NYX* mutations could be responsible for some Chinese high myopia patients without CSNB^22^,^23^.

The forms of CSNB1 and CSNB2 can be differentiated by ERG. However, the correlation of the genotype with the clinical phenotypes “complete” and “incomplete” cannot be established very well^24^. Previous study suggested that patients with CSNB1 mainly had rod-related problems, and patients with CSNB2 had both rod- and cone-related problems, so that the visual acuity on average was better in CSNB1 than in CSNB2. However, the expressivity may be variable within the CSNB1 patients due to the clinical and genetic heterogeneity. Some CSNB1 patients may have dramatically decreased visual acuity, whereas a few patients might have good visual acuity even better than 0.8, and experience only high refractive error and nyctalopia^25^,^26^. In our patients, the patients carrying *NYX* mutation of c.371_377del GCTACCT (p.Y125TfsX138) had reduced visual acuity ≤0.4, myopia of the spherical equivalent range from −3.0D to −10.0D, dissociated vertical deviation, extropia and nystagmus, whereas the patient carrying *NYX* mutation of c.214A>C (p.N72H) had only high myopia of −10.0D and intermittent extropia. Although we cannot establish the correlation of the *NYX* mutations with the clinical phenotype because we don’t have enough patients to be observed, we think that variable expressivity might be related to other genetic or environmental factors rather than to the different mutant types in the same gene.

In summary, we report the clinical characterization of two Chinese families with X-linked CSNB and identified two novel mutations of c. 371_377delGCTACCT and c.214A>C in the *NYX* gene. These two mutations would expand the mutation spectrum of *NYX* and help to study molecular pathogenesis of CSNB.

## Additional Information

**How to cite this article**: Dai, S. *et al.* Two Novel NYX Gene Mutations in the Chinese Families with X-linked Congenital Stationary Night Blindness. *Sci. Rep.* **5**, 12679; doi: 10.1038/srep12679 (2015).

## Figures and Tables

**Table 1:** Clinical features of individuals in Two Chinese families with X-linked CSNB.

Family	ID	Patient/carrier	Age	Mutation	Status	CN,XT,DVD	BCVA OD;OS	Refraction OD;OS
CSNB-01	I:1	patient	78	c. 371_377del	Hemizygous	CN,XT,DVD	0.4; 0.4	−6.75D; −6.75D
CSNB-01	II:4	carrier	53	c. 371_377del	Heterozygous	none	0.8; 0.8	−1.00D; −1.00D
CSNB-01	II:5	carrier	47	c. 371_377del	Heterozygous	none	1.0; 1.0	−0.50D; −0.75D
CSNB-01	III:1	patient	33	c. 371_377del	Hemizygous	CN,XT,DVD	0.2; 0.2	−9.25D; −8.75D
CSNB-01	III:2	carrier	31	c. 371_377del	Heterozygous	none	0.8; 0.8	−1.00D; −1.00D
CSNB-01	III:4	patient	24	c. 371_377del	Hemizygous	CN,XT,DVD	0.4; 0.4	−6.25D; −6.00D
CSNB-01	III:5	patient	22	c. 371_377del	Hemizygous	CN,XT,DVD	0.1; 0.1	−9.75D; −9.50D
CSNB-01	III:6	patient	22	c. 371_377del	Hemizygous	CN,XT,DVD	0.3; 0.3	−5.00D; −4.50D
CSNB-01	IV:1	patient	6	c. 371_377del	Hemizygous	CN,XT,DVD	0.4; 0.4	−3.75D; −3.00D
CSNB-02	III:1	patient	35	c.214A>C	Hemizygous	XT	0.6; 0.6	−9.25D; −9.75D
CSNB-02	III:2	carrier	32	c.214A>C	Heterozygous	none	1.0; 1.0	−1.50D; −2.75D
CSNB-02	IV:1	patient	7	c.214A>C	Hemizygous	XT	0.7; 0.7	−9.00D; −10.00D

CN: congenital nystagmus.

XT: extropia.

DVD: dissociated vertical deviation.

BCVA: Best corrected Visual acuity.

OD: Right eye.

OS: Left eye.
